# Cutaneous Pythiosis in 2 Dogs, Italy

**DOI:** 10.3201/eid2907.230320

**Published:** 2023-07

**Authors:** Andrea Peano, Anna Rita Molinar Min, Alessandra Fondati, Erica Romano, Chiara Brachelente, Ilaria Porcellato, Andrea Amore, Mario Pasquetti

**Affiliations:** Università di Torino, Turin, Italy (A. Peano, A.R. Molinar Min, M. Pasquetti);; Veterinaria Trastevere, Rome, Italy (A. Fondati);; Ospedale Veterinario Gregorio VII, Rome (E. Romano);; Università degli Studi di Perugia, Perugia, Italy (C. Brachelente, I. Porcellato);; Ambulatorio Veterinario Cassia, Cesano, Italy (A. Amore)

**Keywords:** pythiosis, *Pythium insidiosum*, *Pythium periculosum*, dog, lake, freshwater habitats, ecological niche, dermatology, One Health, fungi, parasites, Italy

## Abstract

We report cutaneous pythiosis in 2 dogs in Italy that had recurrent exposure to the same freshwater habitat. Phylogenetic analysis placed the isolates within *Pythium* *insidiosum* complex cluster IV, corresponding to *P*. *periculosum*. In Italy, pythiosis should be considered in differential diagnoses by human and veterinary health professionals.

Pythiosis is a granulomatous disease caused by oomycete organisms affecting mainly horses, humans, and dogs, and is historically associated with humid environments (*1*). In India, where the first cases were reported in horses in the late 19th century, pythiosis is known as bursattee, from burus, signifying rain, because infections appeared during the rainy season (*2*). In other countries, pythiosis is known by different names, such as swamp cancer in Australia and the United States and horse leeches in the United States (*2*).

The genus *Pythium* (kingdom Stramenopila) comprises >120 species inhabiting all soil and wet environments. *Pythium* spp. have a saprophytic life cycle, although many are plant pathogens (*3*). *P.* *insidiosum* has been considered the only species that infects mammals (*2*). In 2003, existence of a cryptic species was suspected on the basis of phylogenetic analyses that indicated *P*. *insidiosum* is a complex with 3 clusters, 1 (cluster III) of which displays substantial divergence from the other 2 clusters (*4*). Recent studies have revealed a fourth cluster (cluster IV); clusters III and IV form a monophyletic group representing a novel species, *P.* *periculosum* (*5*). *P.* *aphanidermatum* has also been reported in 2 cases of human infection (*6*,*7*).

Pythiosis is characterized by the presence of broad, irregular, perpendicular branching hyphae in tissues and cultures that are aseptate in early growth stages or sparsely septate in aged organisms. Biflagellate motile zoospores are the infecting agents, and production in the environment requires free water. The zoospores are attracted by open wounds, encyst on exposed tissue, and develop a germ tube that mechanically penetrates tissue (*2*). The optimal growth temperature range for zoospores is 28°C–37°C (*3*). Pythiosis manifests as rapidly growing granulomatous lesions that might be devastating and life-threatening (*2*). Organs and tissues most affected are the skin, gastrointestinal tract, eyes, and blood vessels (*2*,*3*); disseminated forms are also possible (*3*).

Pythiosis is diffused in warm and humid areas of tropical and subtropical countries, including northeastern Australia, Brazil, Colombia, Costa Rica, India, Thailand, Uruguay, southern and southeastern states of the United States, and Venezuela. The disease has also been reported sporadically in some temperate regions (*3*). A horse with compatible clinical pythiosis features was described in France in 1896 (*8*), whereas confirmed cases have been recently reported in human patients in Spain (*9*,*10*). We describe 2 cases of cutaneous pythiosis in dogs in Italy.

## The Study

**Figure 1:**
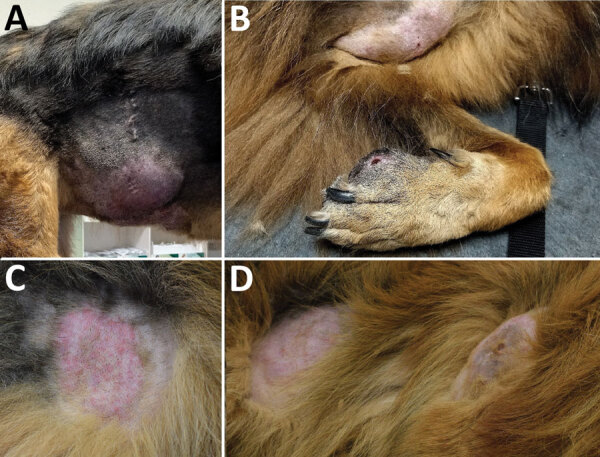
Clinical manifestation of cutaneous pythiosis in 2 dogs, Italy. A, B) Case 1, showing a large mass on the right flank (A) and a large mass on the left axilla and a smaller ulcerated lesion on the first digit of the left forelimb (B). C, D) Case 2, showing detailed image of a plaque (C) and a lateral view of the dog showing 2 plaques on the thorax and thigh (D).

We evaluated soft tissue swellings in 2 unrelated dogs that lived 30 km apart in Rome province of central Italy in October 2022 (case 1) and January 2023 (case 2) (Figure 1). The animals had never been outside of Italy before onset of skin lesions and were otherwise healthy. We collected anamnesis and clinical manifestation data (Table).

**Table:** Anamnesis, clinical history, and manifestations in 2 dogs with cutaneous pythiosis, Italy*

Characteristics	Case 1	Case 2
Date of referral visit	October 2022	January 2023
Breed and age	4-year-old neutered female German Shepherd dog	3-year-old male German Shepherd dog
Life habitat	Dog lived in apartment and periodically swam in the Treja river near home (Rome province) and Lake Bracciano (32 km northwest of Rome), especially during hotter months.	Dog lived in a house with another dog (adult Cane Corso without signs of disease) and had outdoor access to a garden. The dog regularly swam in Lake Bracciano.
Travel history	In summer 2019 and 2020, the dog was taken to northwest Italy (Trentino area), where it occasionally swam in mountain lakes. The dog was never outside of Italy before onset of skin problems.	The dog was never outside of Italy before onset of skin problems.
History of skin problems	Since October 2020, several subcutaneous masses, occasionally ulcerated, developed in different body areas and were surgically removed on different occasions. The last intervention was in May 2022 to remove a large mass on the tail base. A caudectomy was necessary because of massive involvement of tail tissue. Several histopathological evaluations over time showed roughly the same pattern of chronic pyogranulomatous dermatitis and panniculitis involving mastocytes, eosinophils, and neutrophils. Infectious organisms were never reported. The dog was treated with different antimicrobial drugs, glucocorticoids, and cyclosporine, which had no or only partial effect on disease evolution.	In November 2022, an abscess formed on the flexor face of the left elbow and was incised to look for a foreign body (not found). The dog was treated with cephalexin and prednisone, then with clindamycin and prednisone, but showed no response. New lesions developed in other body areas.
Clinical manifestations at referral visit	2 large masses (≈10 cm × 12 cm × 10 cm) on the left axilla and right flank; a 3rd smaller mass with a small ulcer on the first digit of the left forelimb.	Multiple dermal and subcutaneous papules and plaques of variable size, some with fistulae yielding serosanguineous exudate.

*Clinical manifestations are shown for both dogs (Figure 1).

**Figure 2:**
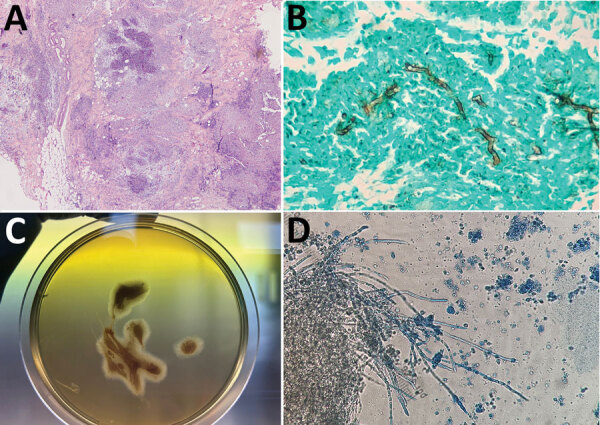
Histological analyses of cutaneous pythiosis in 2 dogs, Italy. A) Section from punch biopsy of cutaneous mass showing confluent pyogranulomatous and eosinophilic granulomas with central necrosis in the deep dermis and panniculus. Hematoxylin and eosin stain; original magnification ×100. B) Section from punch biopsy of cutaneous mass showing broad, irregular branching hyphae. Grocott methenamine silver stain; original magnification ×400. C) Culture (24-hour) of an aspirate from a mass found in case 2. Aspirate was cultured on Sabouraud dextrose agar without antimicrobial drugs, and showed submerged, colorless colonies with irregular radiate patterns. D) Microscopic appearance of the colonies indicating broad (4–10 µm in diameter), hyaline, and sparsely septate hyphae. Original magnification ×250.

Histopathologic results of lesion punch biopsies showed multifocal to coalescing (pyo)granulomatous and eosinophilic dermatitis and panniculitis with intralesional, irregular branching hyphae (Figure 2, panel A). The hyphae were positive for Grocott methenamine silver stain, appearing dark brown (Figure 2, panel B), whereas they were negative after staining with periodic acid Schiff stain. Culturing on Sabouraud dextrose agar (for fungi) containing chloramphenicol and gentamycin yielded negative results.

To obtain a definitive identification, we extracted genomic DNA from biopsied tissues by using a NucleoSpin Tissue kit (Macherey-Nagel, https://www.mn-net.com). We PCR amplified and sequenced the internal transcribed spacer (ITS) region by using the primer pair ITS4 and ITS5 (*11*). We obtained identical sequences (GenBank accession nos. OQ532907 [case 1] and OQ532908 [case 2]) that were closely related to ITS sequences of *P*. *insidiosum* from GenBank (compared by using BLASTn, https://blast.ncbi.nlm.nih.gov). We aligned sequences belonging to different clusters within the *P*. *insidiosum* complex (*5*) by using MEGA11 software (https://www.megasoftware.net). We conducted phylogenetic analysis by using the neighbor-joining method (bootstrap analysis with 1,000 replicates); sequences obtained from the infected dog tissues clustered with *Pythium* cluster IV (Appendix Figure), corresponding to the newly described species *P*. *periculosum* (*5*).

Although an environmental study would be necessary for confirmation, Lake Bracciano near Rome might have been the infection source (Table). Both dogs regularly swam in the lake, which has vast stagnant waters with stable temperatures of ≈30°C in hotter months, features known to support pathogenic *Pythium* spp. growth (*2*,*3*).

Additional material was collected from the lesion in case 2 through fine-needle aspiration. We suspected the organism failed to grow in previous cultures because of chloramphenicol supplementation (*10*). Therefore, we cultured the aspirate on unsupplemented Sabouraud dextrose agar at 37°C. After a 24-hour incubation, submerged, colorless colonies with irregular radiate patterns developed from the aspirated material (Figure 2, panel C). Microscopically, the hyphae were broad (4–10 µm in diameter), hyaline, and sparsely septate (Figure 2, panel D). Colonies were identified as *P*. *periculosum* by using the same molecular approach applied to biopsies.

## Conclusions

Our cases suggest a geographic distribution of pythiosis broader than previously recognized, aligning with other reports of the disease outside of classical tropical and subtropical areas, such as US regions near the border of Canada, as well as Spain, Israel, Japan, and South Korea (*2*,*3*). Because the average climatic conditions in temperate zones appear unsuitable for the causative oomycete life cycle, cases in those areas likely occur in restricted ecologic niches. Our report illustrates that concept; central Italy has a primarily Mediterranean climate that has mild, sometimes rainy winters and sunny, hot, and usually dry summers. Those features do not fit the description of areas more prone to pythiosis. However, the zone where we suspect the dogs became infected has features of a pythiosis-risk area (warm, humid environment). Increasing reports of pythiosis in nonendemic regions might indicate an expansion of the causative oomycetes because of global climate changes. Another interpretation is that more cases are recognized and published because of increased awareness of healthcare personnel and availability of diagnostic tools (*3*). Environmental and clinical studies will be necessary to address those hypotheses.

The dogs in this report showed signs typically associated with the cutaneous form of pythiosis, including developing multiple masses or dermal plaques over time (*3*). Gastrointestinal pythiosis in dogs is another form of the disease characterized by weight loss, vomiting, diarrhea, and hematochezia (*3*,*12*).

Genetic variation associated with geographic provenance exists for species within the *P. insidiosum* complex (*4*,*5*). *P*. *insidiosum* cluster I is mainly found throughout the Americas, whereas *P*. *insidiosum* cluster II is typically found in Asia and Australia. *P. periculosum* cluster III has been reported mainly in the United States and is sympatric with members of cluster I (*5*). The genotype identified in our cases (*P. periculosum* cluster IV) seems to have a broader distribution; most reports of human cases have been from Thailand and India (*3*). However, *P. periculosum* cluster IV has also been recovered from environmental samples in Brazil and the United States (*13*,*14*) and from human patients in Israel (*15*) and Spain (*10*), which is noteworthy because of the close proximity of Spain to Italy.

In conclusion, from a One Health perspective, our study shows the environmental presence of an unexpected, exotic pathogen that could potentially infect humans in a country with a temperate climate. In Italy, pythiosis should now be considered a differential diagnosis by human and veterinary health professionals, especially in cases where there is a history of exposure to freshwater habitats.

Appendix Additional information for cutaneous pythiosis in 2 dogs, Italy.
