# Supplementary material for: Cutaneous Pythiosis in 2 Dogs, Italy
Source: Emerg Infect Dis. 2023 Jul;29(7):1447–50. doi: 10.3201/eid2907.230320 (PMC10310393; doi:10.3201/eid2907.230320)
Supplement: Appendix — Additional information for cutaneous pythiosis in 2 dogs, Italy. [file 23-0320-Techapp-s1.pdf]

Article DOI: https://doi.org/10.3201/eid2907.230320

EID cannot ensure accessibility for supplementary materials supplied by authors. Readers who have difficulty accessing supplementary content should contact the authors for assistance.

Cutaneous Pythiosis in 2 Dogs, Italy

Appendix

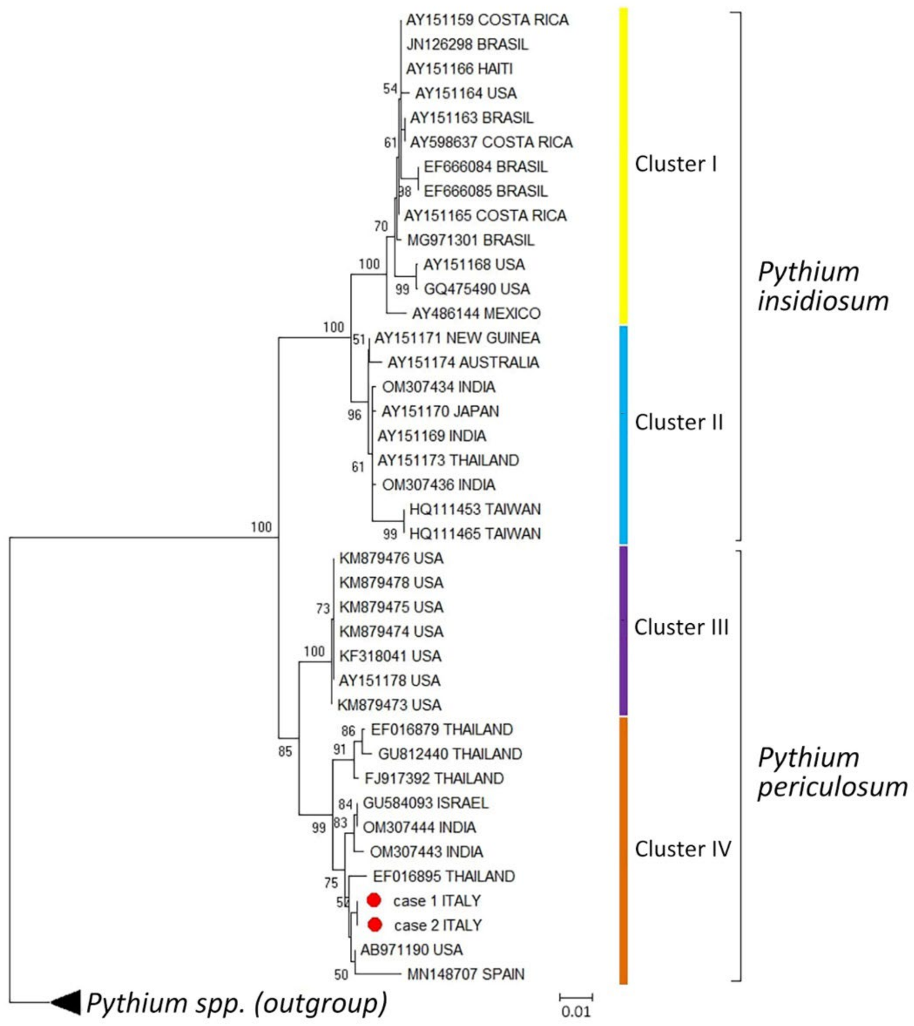

Appendix Figure. Phylogenetic analysis of rDNA internal transcribed spacer sequences from isolates collected from cutaneous pythiosis lesions from 2 dogs in Italy. Tree was constructed by using the neighbor-joining method. Sequences obtained from lesion biopsies from the 2 dogs in Italy are highlighted by red circles. Sequences for comparison were obtained from GenBank and include those from isolates belonging to the 4 clusters recognized within the *Pythium insidiosum* complex. Scale bar indicates nucleotide substitutions per site.
